# Genomic alterations of ground-glass nodular lung adenocarcinoma

**DOI:** 10.1038/s41598-018-25800-2

**Published:** 2018-05-16

**Authors:** Hyun Lee, Je-Gun Joung, Hyun-Tae Shin, Duk-Hwan Kim, Yujin Kim, Hojoong Kim, O. Jung Kwon, Young Mog Shim, Ho Yun Lee, Kyung Soo Lee, Yoon-La Choi, Woong-Yang Park, D. Neil Hayes, Sang-Won Um

**Affiliations:** 1 Division of Pulmonary and Critical Care Medicine, Department of Medicine, Samsung Medical Center, Sungkyunkwan University School of Medicine, Seoul, Korea; 2 Samsung Genome Institute, Samsung Medical Center, Seoul, Korea; 3 Department of Molecular Cell Biology, Sungkyunkwan University School of Medicine, Suwon, Korea; 4 Department of Thoracic and Cardiovascular Surgery, Samsung Medical Center, Sungkyunkwan University School of Medicine, Seoul, Korea; 5 Department of Radiology and Center for Imaging Science, Samsung Medical Center, Sungkyunkwan University School of Medicine, Seoul, Korea; 6 Department of Pathology and Translational Genomics, Samsung Medical Center, Sungkyunkwan University School of Medicine, Seoul, Korea; 7 Lineberger Comprehensive Cancer Center, University of North Carolina at Chapel Hill, Chapel Hill, North Carolina USA

## Abstract

In-depth molecular pathogenesis of ground-glass nodular lung adenocarcinoma has not been well understood. The objectives of this study were to identify genomic alterations in ground-glass nodular lung adenocarcinomas and to investigate whether viral transcripts were detected in these tumors. Nine patients with pure (n = 4) and part-solid (n = 5) ground-glass nodular adenocarcinomas were included. Six were females with a median age of 58 years. We performed targeted exon sequencing and RNA sequencing. EGFR (n = 10), IDH2 (n = 2), TP53 (n = 1), PTEN (n = 1), EPHB4 (n = 1), and BRAF (n = 1) were identified as driver mutations by targeted exon sequencing. Vasculogenesis-associated genes including NOTCH4 and TGFBR3 expression were significantly downregulated in adenocarcinoma tissue versus normal tissue (adjusted P values < 0.001 for both NOTCH4 and TGFBR3). In addition, five novel fusion gene loci were identified in four lung adenocarcinomas. However, no significant virus-associated transcripts were detected in tumors. In conclusions, EGFR, IDH2, TP53, PTEN, EPHB4, and BRAF were identified as putative driver mutations of ground-glass nodular adenocarcinomas. Five novel fusion genes were also identified in four tumors. Viruses do not appear to be involved in the tumorigenesis of ground-glass nodular lung adenocarcinoma.

## Introduction

With the increased use of chest computed tomography (CT), ground-glass nodules (GGNs), which are defined as a slight increase in density that do not obscure the underlying vascular or bronchial structures^1,2^, are increasingly encountered in clinical practice. Histological subtypes of GGNs include both pre-invasive lesions such as atypical adenomatous hyperplasia and early stage lung adenocarcinoma including adenocarcinoma *in situ*, minimally invasive adenocarcinoma, and lepidic-predominant invasive adenocarcinoma^3,4^. GGNs generally grow slowly and have a good prognosis^2^. Patients with ground-glass opacity (GGO) components of more than 50% show reduced vascular invasion and less lymph node metastasis than those with components of less than 50%^5^.

Lung adenocarcinoma is the most common subtype of non-small cell lung cancer (NSCLC)^6,7^. Understanding the tumorigenesis of ground-glass nodular lung adenocarcinoma is crucial for the proper management of early stage NSCLC. However, in-depth molecular pathogenesis of this disease has not been well understood. Ovine pulmonary adenocarcinoma (OPA) is a transmissible lung tumor of sheep caused by the jaagsiekte sheep retrovirus (JSRV)^8^. Ground-glass nodular lung adenocarcinoma may be related to this viral infection through a similar histology with OPA^8,9^. Furthermore, JSRV was detected in human lung cancer tissue arrays^10^. There has been suspicion that multiple ground-glass nodular lung adenocarcinomas in the same patient may be the result of bronchogenic spread from a viral infection rather than from hematogenous metastasis^11^.

The purpose of this study was to identify genomic changes in ground-glass nodular lung adenocarcinomas using targeted exon sequencing and RNA sequencing. We further investigated whether viral transcripts were identified in these tumors.

## Results

### Patients

**Figure 1:**
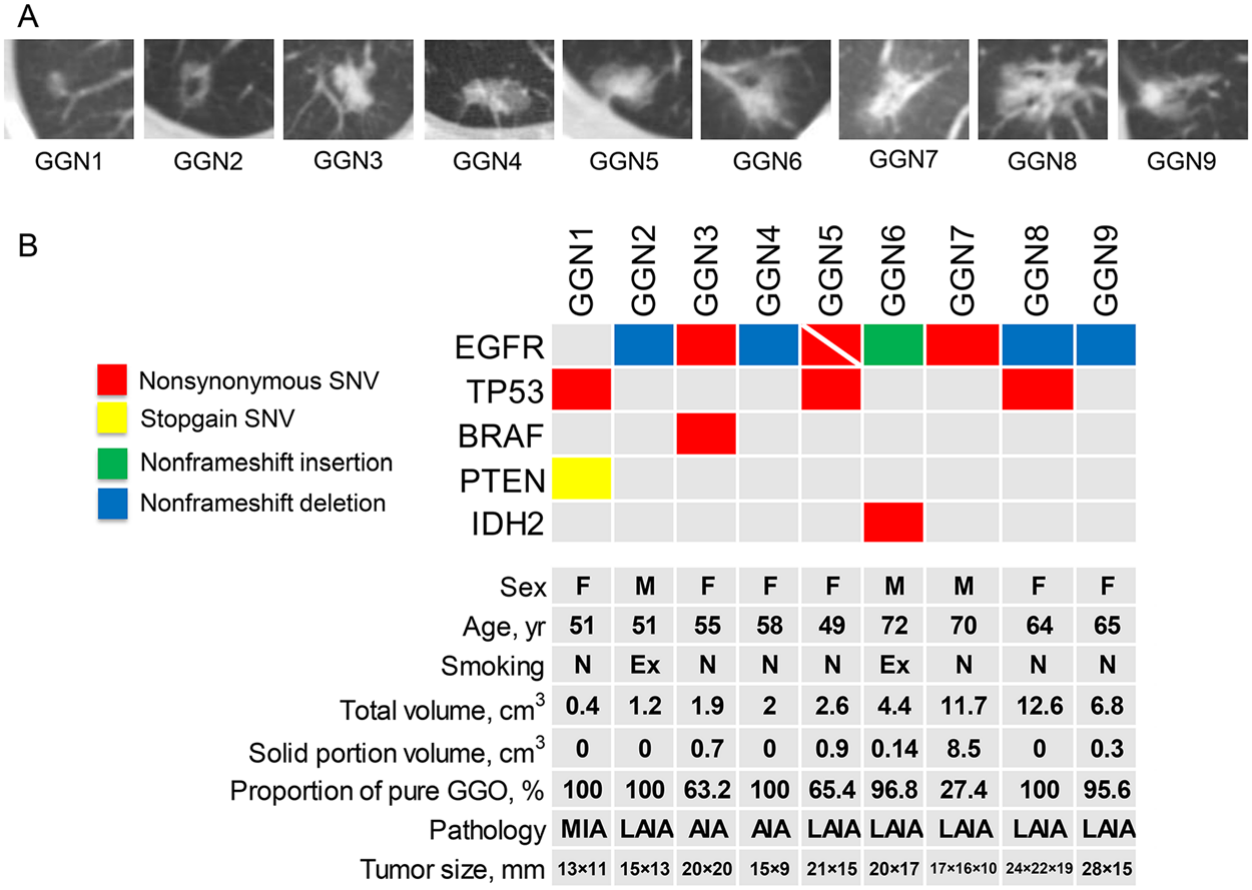
(**A**) CT images of nine GGNs and (**B**) summary of mutations and clinical characteristics of nine patients with ground-glass nodular lung adenocarcinoma. Variable allele frequency for each mutation was as follow; 0.116 for TP53 R158L, 0.088 for PTEN Q245X and 0.059 for EPHB4 S703C in GGN1, 0.019 for EGFR p.745_750del in GGN2, 0.385 for EGFR L858R and 0.299 for BRAF W604G in GGN3, 0.095 for EGFR p.745_750del in GGN4, 0.465 for EGFR L858R and 0.238 for EGFR R776S in GGN5, 0.111 for EGFR p.A767delinsASVD, 0.060 for IDH2 P172L, and 0.060 for IDH2 K140N in GGN6, 0.095 for EGFR L858R in GGN7, 0.370 for EGFR A750P and 0.387 for EGFR p.746_748del in GGN8, and 0.053 for EGFR p.746_751del in GGN9. CT, computed tomography; GGN, ground-glass nodule; F, female; M, male; N, never-smoker; Ex, ex-smoker; SNV; single nucleotide variant; MIA, minimally invasive adenocarcinoma; LAIA, lepidic and acinar predominant invasive adenocarcinoma; AIA, acinar predominant invasive adenocarcinoma.

The baseline and clinical characteristics of nine patients with ground-glass nodular lung adenocarcinoma are summarized in Table 1 and Fig. 1. There were six females (66.7%) and three males (33.3%) with a median age of 58 years (IQR, 51–68 years). Seven subjects (77.8%) were never-smokers and two (22.2%) were ex-smokers. Four subjects (44.4%) had pure GGNs and five (55.6%) had part-solid GGNs (Fig. 1A). The median tumor volume of GGNs measured by chest CT scan was 2.6 cm3 (IQR, 1.6–9.3 cm^3^). The pathologic classification of the nine ground-glass nodular adenocarcinomas included invasive adenocarcinoma (lepidic and acinar predominant type, n = 6), invasive adenocarcinoma (acinar predominant type, n = 2), and minimally invasive adenocarcinoma (non-mucinous type, n = 1).

**Table 1:** Baseline and clinical characteristics of nine patients with ground-glass nodular lung adenocarcinoma.

	No (%) or median (IQR)
Age, years	58 (51–68)
Female	6 (66.7)
Smoking history	
Never-smoker	7 (77.8)
Ex-smoker	2 (22.2)
Ground-glass nodule	
Total volume, cm^3^	2.6 (1.6–9.3)
Pure	4 (44.4)
Part-solid	5 (55.6)
Pathologic classification	
Minimally invasive adenocarcinoma (nonmucinous)	1 (11.1)
Invasive adenocarcinoma (lepidic and acinar predominant)	6 (66.7)
Invasive adenocarcinoma (acinar predominant)	2 (22.2)
Surgery type	
Lobectomy	7 (77.8)
Wedge resection	1 (11.1)
Segmentectomy	1 (11.1)
Follow-up duration, months	50.8 (42.0–56.7)
Relapse	0 (0)
Survival	9 (100)

Data are presented as median (IQR) or median (%).

IQR, interquartile range.

### Targeted exon sequencing

Approximately 82% of total mapped reads (an average of 41,314,301 reads) with a mean depth coverage of 978.6× were obtained from nine ground-glass nodular lung adenocarcinoma genomes, and approximately 99% of the target regions were covered sufficiently, at least 100× regions. A total of 16 somatic DNA changes were identified as driver mutations in nine ground-glass nodular adenocarcinomas including non-synonymous single nucleotide variant (SNV) (n = 10), nonframeshift deletion (n = 4), nonframeshift insertion (n = 1), and stopgain SNV (n = 1) (Fig. 1B and Supplementary Table S3). Of 6 somatic DNA changes, 10 epidermal growth factor receptor (EGFR) mutations were detected in eight lung adenocarcinoma samples (Fig. 1B and Table 2). The subtypes of 10 EGFR mutations are summarized in Table 2. Two EGFR mutations were found in GGN5 (R776S in exon 20 and L858R in exon 21) and GGN8 (p.746_748del and A750P in exon 19). EGFR mutation was the only genomic change in GGN2, GGN4, GGN5, GGN7, GGN8, and GGN9. EGFR mutation was detected with other mutations including proto-oncogene B-Raf (BRAF) in GGN3 (W604G in exon15) and isocitrate dehydrogenase 2 (IDH2) in GGN6 (P172L and K140N in exon4). GGN1 without EGFR mutations had TP53 (R158L in exon5), phosphatase and tensin homolog (PTEN) (Q245X in exon 7), and EPH receptor B4 (EPHB4) (S703C in exon 12) mutations.

**Table 2:** Subtypes of EGFR mutations detected by targeted exon sequencing.

Exon	Amino acid change	GGN	Total (*N* = 8)
19			4 (50.0)
	p.745_750del	GGN2, GGN4	2 (25.0)
	p.746_748del0	GGN8^*^	1 (12.5)
	A750P	GGN8^*^	1 (12.5)
	p.746_751del	GGN9	1 (12.5)
20			2 (25.0)
	R776S	GGN5^†^	1 (12.5)
	p.A767delinsASVD	GGN6	1 (12.5)
21			3 (37.5)
	L858R	GGN3, GGN5^†^, GGN7	3 (37.5)

Data are presented as numbers (%).

^*^GGN8 had two EGFR mutations (A750P and p.746_748del).

^†^GGN5 had two EGFR mutations (L858R and R776S).

GGN, ground-glass nodule; EGFR, epidermal growth factor receptor.

### Whole transcriptome analysis

Approximately 89.6% and 86.6% of total reads were mapped sufficiently in lung adenocarcinoma samples and normal samples, respectively. The unique aligned reads among total reads were approximately 85.5% and 82.1% for lung adenocarcinoma samples and normal samples, respectively (Supplementary Table S4).

### Clustering analysis with differentially expressed genes and functional analysis

**Figure 2:**
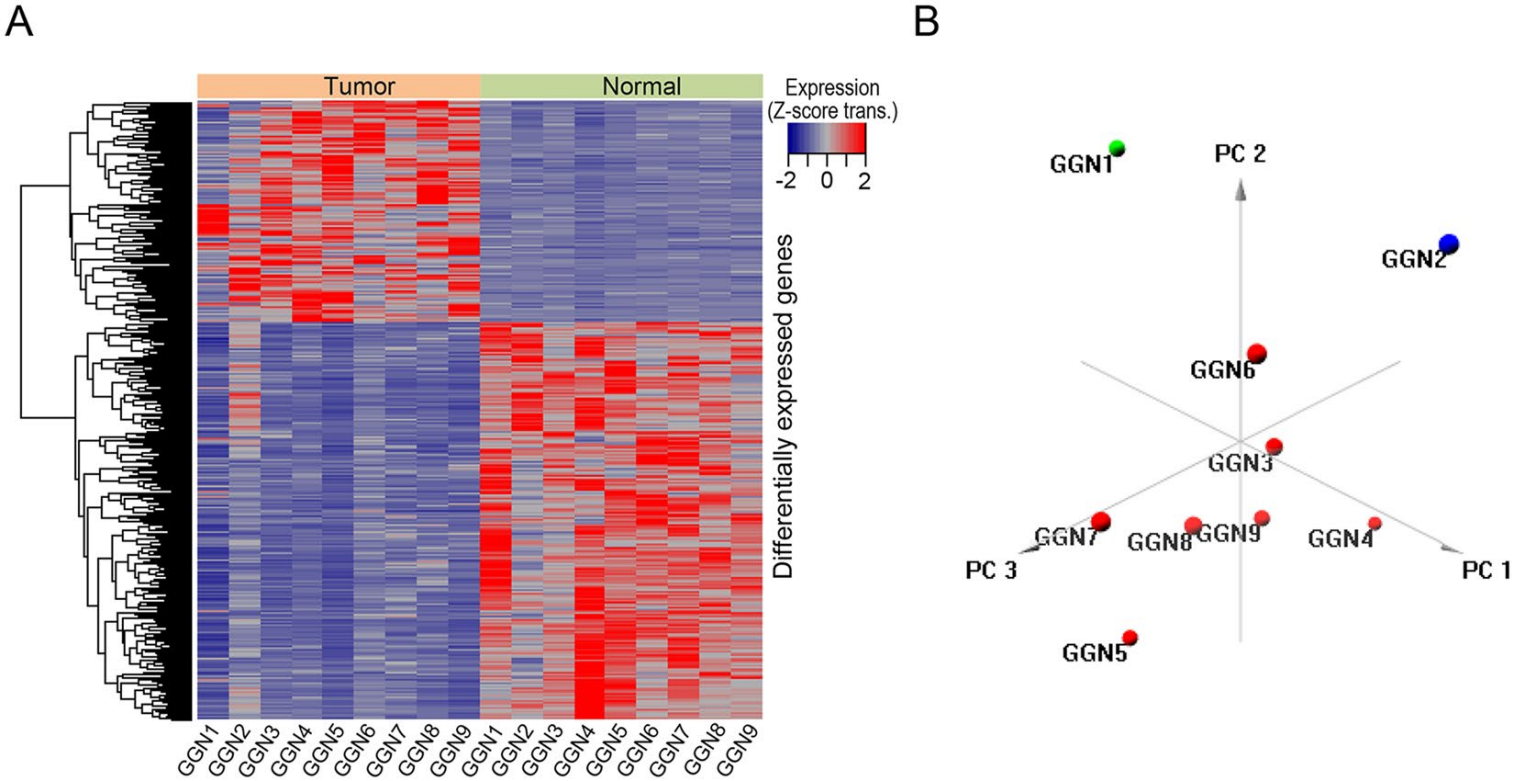
(**A**) Hierarchical clustering of lung adenocarcinoma with differentially expressed genes and (**B**) principal component analysis. GGN1-2 had differently expressed profiles compared to GGN 3–9 in hierarchical clustering and principal component analysis. GGN, ground-glass nodule.

**Figure 3:**
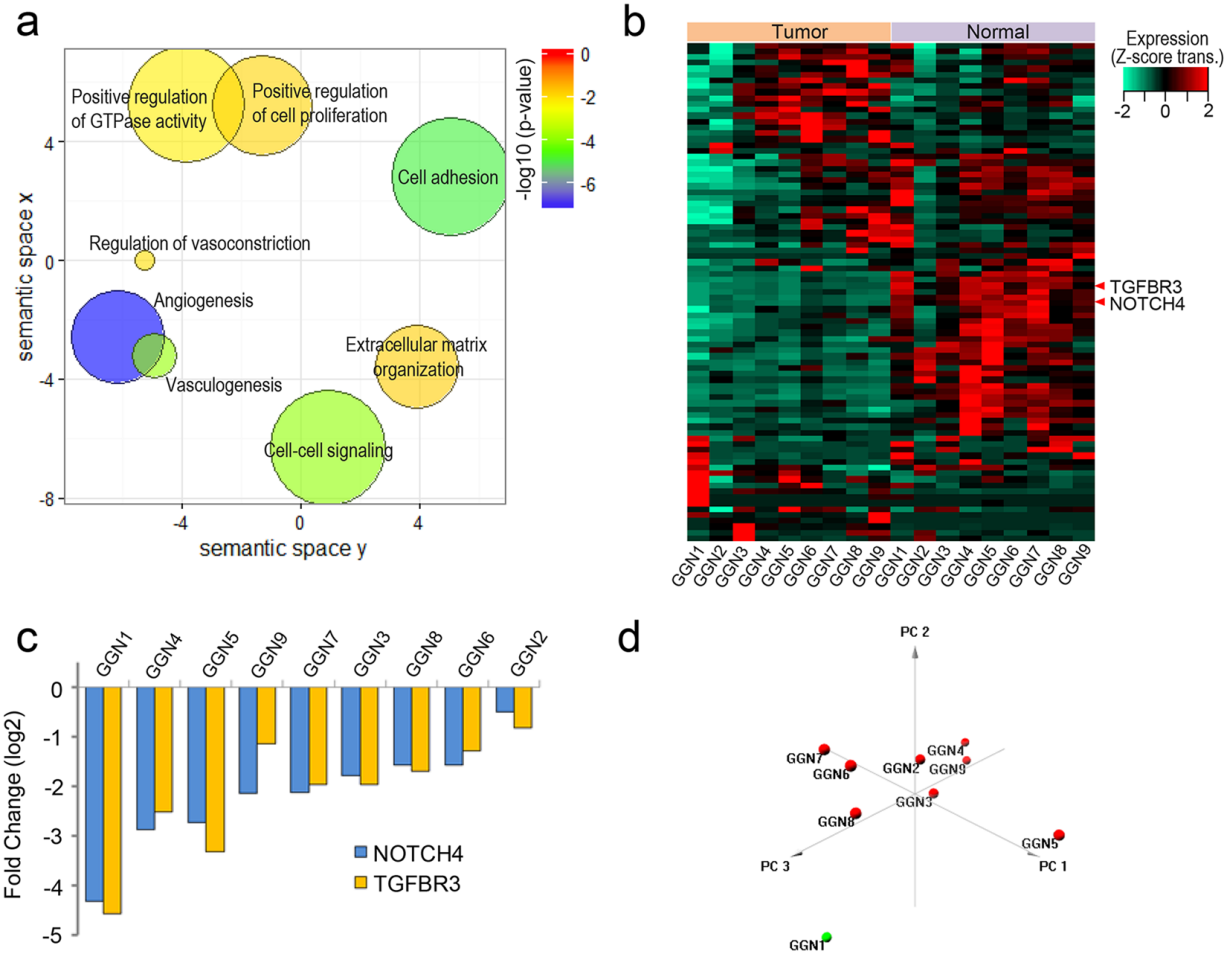
Functional analysis of gene expression. (**a**) Semantic similarity-based scatter plots of enriched gene ontology terms with significantly altered genes, (**b**) heatmap of vasculogenesis-associated genes, (**c**) expression ratios of NOTCH4 and TGFBR3, and (**d**) principal component analysis of vasculogenesis-associated genes. (**a**) The downregulated genes were enriched for vasculogenesis (p = 1.27E^−4^). The more reddish color is the higher expression of a certain gene set. (**b**) Of the vasculogenesis-associated genes, NTOCH4 and TGFBR3 expression was significantly downregulated in adenocarcinoma tissue versus normal tissue (adjusted p < 0.001 for NOTCH4 and adjusted p < 0.001 for TGFBR3). (**c**) Notable downregulation of NOTCH4 and TGFBR3 was detected in GGN1. (**d**) Principal component analysis of vasculogenesis-associated genes showed that only GGN1 was an outlier. GGN, ground-glass nodule; NOTCH4, neurogenic locus notch homolog protein 4; TGFBR3, transforming growth factor beta receptor 3.

**Figure 4:**
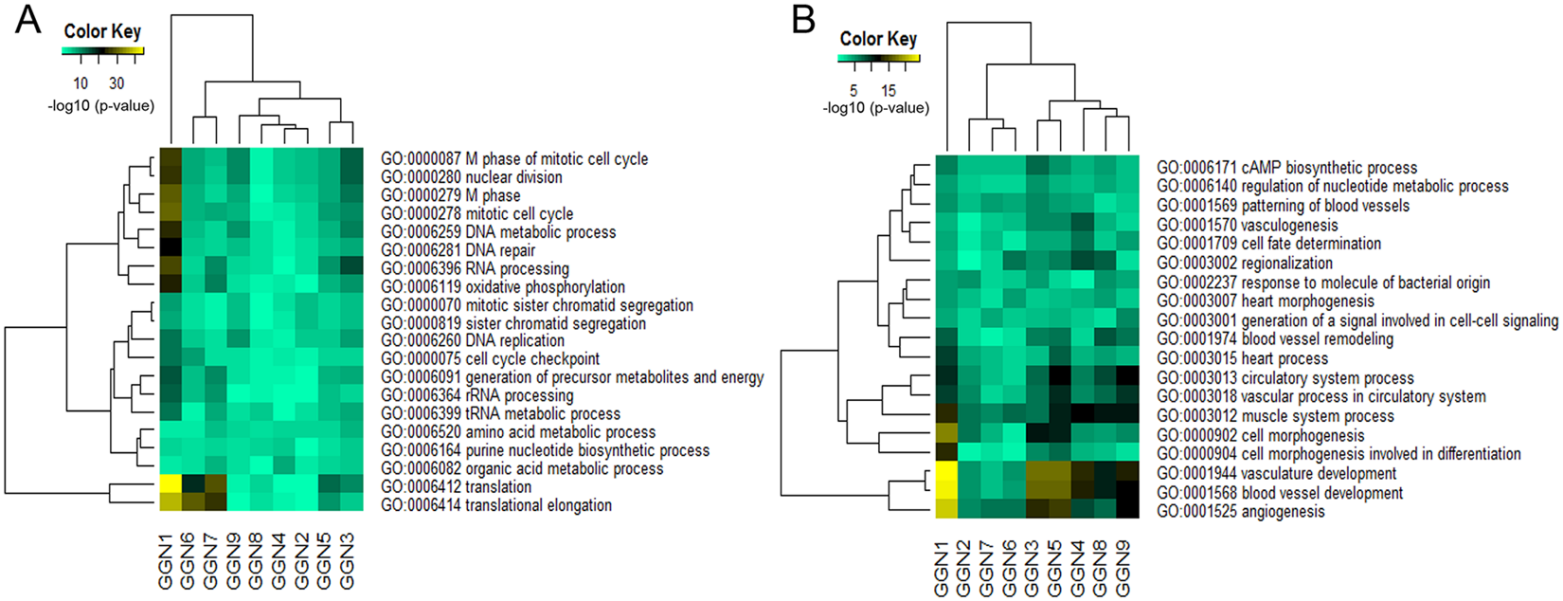
Heatmap of gene ontology terms enriched for individual GGNs with (**A**) upregulated genes and (**B**) downregulated genes. GGN1 had significantly upregulated genes associated with translation and cell cycles (**A**) and significantly downregulated genes associated with angiogenesis (**B**) compared to the other GGNs. GGN, ground-glass nodule.

A total of nine pairs of samples in nine patients were included for clustering analysis. Patients were grouped together with 643 differentially expressed genes in lung adenocarcinoma tissue versus normal tissue (adjusted P value < 0.01) (Fig. 2A,B and Supplementary Table S5). Hierarchical clustering showed that GGN1 without EGFR mutations had a distinct gene expression profile compared to other GGNs with EGFR mutations (Fig. 2A). Principal component analysis showed that two GGNs (GGN1 and GGN2) with a smaller tumor volume (<1.5 cm^3^) and 100% pure GGO had differently expressed profiles compared to seven GGNs (GGN3–9) with a larger tumor volume (>1.5 cm^3^) (Figs 1A,B and 2B). Functional enrichment analysis of the gene sets showed that the downregulated genes were significantly enriched for vasculogenesis (p = 1.27E^−4^) (Fig. 3a). Vasculogenesis-associated genes including neurogenic locus notch homolog protein 4 (NOTCH4) and transforming growth factor beta receptor 3 (TGFBR3) expression were significantly downregulated in adenocarcinoma tissue versus normal tissue (adjusted p < 0.001 for NOTCH4 and adjusted p < 0.001 for TGFBR3) (Fig. 3b). Notable downregulation of NOTCH4 and TGFBR3 was detected in GGN1 (Fig. 3c). GGN1 was an outlier in principal component analysis (Fig. 3d). We also performed gene set enrichment analysis for each GGN. GGN1 had significantly upregulated genes associated with translation and cell cycles compared to the other GGNs (Fig. 4A). GGN1 also had significantly downregulated genes associated with vasculogenesis compared to the other GGNs (Fig. 4B).

### Detection of fusion genes

A total of five fusion gene loci were detected in four GGNs using the TopHat fusion tool as follows (Table 3 and Supplementary Fig. S2): mediator complex subunit 13-like (MED13L) → tudor domain containing 3 (TDRD3) in GGN3, sterile alpha motif domain containing 12 (SAMD12) → TATA-box binding protein associated factors (TAF2) in GGN4, centrosome-associated protein 350 (CEP350) → topoisomerase (DNA) II alpha (TOP2A) and transcriptional adaptor 2A (TADA2A) → matrix metallopeptidase 9 (MMP9) in GGN8, and transmembrane protein 243 (TMEM243) → cyclin D binding myb-like transcription factor 1 (DMTF1) in GGN9. The fusion genes detected by RNA sequencing were all validated by RT-PCR (Supplementary Fig. S3).

**Table 3:** Fusion gene loci identified in four patients.

GGN	Fusion gene	Frame shift class	Gene 1			Gene 2			Fusion junction sequence
			Gene 1	Ch	Position	Gene 2	Ch	Position	
GGN3	MED13L → TDRD3	Inframe	MED13L	12	116403887	TDRD3	13	61141659	GCTCAAAACCAGTGCCCTCTCTTCTTAAAGgaggaagaaggcacctacgatcaaactctg
GGN4	SAMD12 → TAF2	Frame shift	SAMD12	8	119592954	TAF2	8	120797506	CGACTGCAGGCAGAAGCTGAGACGGCTAAGgaataaaaagaaaaaaatcccactgatgaa
GGN8	CEP350 → TOP2A	Inframe	CEP350	1	179924277	TOP2A	17	38568070	GGTAGCCGAGGGCGGAGGCGACACTCTCAGgtaaaaggatttcgtagttatgtggacatg
	TADA2A → MMP9	Inframe	TADA2A	17	35802753	MMP9	20	44638505	TACATGCCAGCTCGAGCAGATTTCATTGAGgaatacctgtaccgctatggttacactcgg
GGN9	TMEM243 → DMTF1	Frame shift	TMEM243	7	86848742	DMTF1	7	86800311	TCACTTCAAAGCTCTGATCATTTTCTGAAActtggcggacgtctccccaaacagaggtct

GGN, ground glass nodule; Ch, chromosome.

### Detection of viral genomes in ground-glass nodular lung adenocarcinoma

We evaluated whether viral transcripts were detected in lung adenocarcinoma tissues. A total of 6,251 viral genomes were evaluated, but no enriched viral transcripts were detected in the tissues of lung adenocarcinoma.

## Discussion

This study investigated genomic alterations in nine ground-glass nodular lung adenocarcinomas using targeted exon sequencing and whole transcriptome analysis. We identified EGFR, IDH2, TP53, PTEN, EPHB4, and BRAF as potential driver mutations in ground-glass nodular lung adenocarcinomas. In addition, 643 genes were differentially expressed in lung adenocarcinoma tissue versus normal tissue with predominant downregulation of vasculogenesis-associated genes such as NOTCH4 and TBFBR3. We also identified five novel fusion gene loci in four ground-glass nodular adenocarcinomas. However, viral transcripts were not detected in the tumor tissues. To the best of our knowledge, this is the first study to investigate whether viral transcripts were identified in ground-glass nodular lung adenocarcinoma using RNA sequencing.

In agreement with previous studies of early lung adenocarcinoma, EGFR mutations are major driver mutations in this study^12,13^. However, the frequency of EGFR mutation in this study (89%) was higher than those of previous studies (30–40% in Asian patients with early lung adenocarcinoma)^12,13^, for several possible reasons. First, previous studies have shown that ground-glass nodular lung adenocarcinoma has more frequent EGFR mutations (up to about 63%) compared to other types of adenocarcinoma^13,14^. Second, a highly sensitive targeted exon sequencing method was used in this study. Finally, the differences may be explained by the different study populations, as 67% of our study subjects were Asian females with no history of smoking.

BRAF is a proto-oncogene that encodes a serine/threonine kinase and is identified in about 0.8–8% of lung adenocarcinoma^15^. It has been reported that BRAF mutations occur exclusively with EGFR, K-RAS, and EML4/ALK translocations^15,16^. However, a recent study also showed that the BRAF mutation can be concurrent with the EGFR mutation, as in our study^17^. GGN1 not harboring EGFR mutation, has mutations in two tumor suppressor genes (PTEN and TP53) and one oncogene (EPHB4); TP53 is a tumor suppressor gene which can act as a driver mutation. In a previous study, TP53 was the most frequent mutation in EGFR/KRAS/ALK-negative lung adenocarcinoma in never-smokers^18^. PTEN is a tumor suppressor gene which has been shown to be associated with both NSCLC and SCLC^19–21^. Loss of the PTEN protein is a common event in early stage NSCLC cell lines^19^. EPHB4 is a member of EPH family of receptor tyrosine kinase, which is associated with cellular proliferation and motility in lung cancer^22^. EPHB4 mutation is mutually exclusive to EGFR mutation in lung cancer as in our study^22^. These results suggest that PTEN, TP53, and EPHB4 might be involved in the EGFR-independent early tumorigenesis of ground-glass nodular lung adenocarcinoma^23^. IDH2 is a metabolism-associated gene, which might act as oncogene by promoting cancer cell metabolism and growth. A recent study showed that functional IDH2 variant might be associated with increased risk of lung cancer development^24^.

Using whole transcriptome analysis, we identified that vasculogenesis-associated genes such as NOTCH4^25^ and TGFB3^25^ were down-regulated. The downregulation of vasculogenesis-associated genes could be associated with a good prognosis and may explain the indolent clinical characteristics of ground-glass nodular lung adenocarcinoma. The histological subtype of GGN1, which had TP3, PTEN, and EPHB4 mutations, was a minimally invasive adenocarcinoma (invasive component ≤5 mm in pathology specimen)^26^. GGN1 had a distinct gene expression profile compared to the other GGNs (invasive adenocarcinoma) with EGFR mutations in this study. GGN1 had significantly upregulated genes associated with translation and cell cycles (Fig. 4A) and significantly downregulated genes associated with angiogenesis (Fig. 4B), compared to the other GGNs.

Whole transcriptome analysis also revealed five novel fusion gene loci (MED13L/TDRD3, SAMD12/TAF2, CEP350/TOP2A, TADA2A/MMP9, and TMEM243/DMTF1) in four patients. MED13L encodes a subunit of the mediator complex that functions as a transcriptional coactivator for nearly all of the RNA polymerase II-dependent genes, which are required for Rb/E2F-mediated inhibition of cell proliferation^27^. TDRD3 acts as an effector promoting transcription by promoting histone tail methylation^28^. The overexpression of SAMD9 suppresses tumorigenesis of NSCLC *in vitro*^29^. TAFs form the RNA polymerase II initiation factor and contribute towards the regulation of dedifferentiation states in ovarian cancer. TAF2 increases copy number or mRNA expression in ovarian cancer^30^. CEP350 is a large protein containing a CAP-Gly domain, which localizes to the centrosome. CEP acts as a tumor-suppressor gene in human melanomas^31^. TOP2A is commonly altered at both the gene copy number and gene expression level in cancer cells and has been suggested to play an important role in chromosome instability in human cancers^32^. TADA2A is related to chromatin organization and RNA polymerase II transcription initiation and promoter clearance^33^. Dysregulation of MMP has been shown to be associated with cancer cell progression, migration, and invasion through the basement membrane^34^. High-level MMP9 activity is correlated with aggressive tumor behaviors and poor clinical outcomes in early stage lung adenocarcinoma after complete resection^35^ TMEM243 is an transmembrane protein and overexpression was associated with resistance to paclitaxcel^36^. DMTF1 plays as a mediator of RAS signaling to induce cell-cycle arrest. DMTF1 is hemizygously deleted in about 40% of NSCLC^37^. The fusion genes identified in this study are novel and could play a role in tumor progression rather than initiation, as the five fusion genes were mainly detected in relatively larger tumors (GGN3, 4, 8, and 9). However, they have not been functionally validated. Therefore, further studies are needed to elucidate the role of the fusion genes detected in this study.

In this study, we identified several putative driver mutations, gene expression changes, and fusion genes in patients with ground-glass nodular adenocarcinoma. However, it is a big challenge to prioritize the functional importance of the genomic alterations. In order to understand how genomic alterations drive cancer cell survival and proliferation, integrated network analysis of data obtained from multiple omics could be helpful^38,39^. For example, a previous study showed that the basal subtype of breast cancer has its unique cell subtype-specific signaling pathway (p53 and genome instability), in addition to commonly exploited primitive core signaling pathways in breast cancer^39^. The present study showed that the core-signaling pathway in the development of ground-glass nodular adenocarcinoma is EGFR mutation. However, as shown in breast cancer, the tumorigenesis cannot be fully explained by core signaling pathways^39^. Therefore, in order to elucidate underlying signaling mechanisms governing cancer cell survival and proliferation, it is crucial to perform an analysis that integrates genomic alteration information and functional genetic data in the future.

In summary, EGFR mutations were detected in eight of nine tumors and are supposed to be a dominant driver mutation for ground-glass nodular lung adenocarcinoma. However, the smallest GGN (minimally invasive adenocarcinoma) had PTEN, TP53, and EPHB4 mutations without EGFR mutations. The combination of tumor suppressor genes (PTEN and TP53) and oncogene (EPHB4) may play a role in the early tumorigenesis of ground-glass nodular adenocarcinoma. Fusion genes are detected in relatively larger tumors and could be associated with tumor progression rather than initiation. The downregulation of vasculogenesis-associated genes could explain the indolent clinical characteristics of ground-glass nodular adenocarcinoma such as reduced vascular invasion and less lymph node metastasis. GGNs with a more solid component (GGN3 for 37% and GGN7 for 73%) had a higher expression of NOTCH4 and TGFBR3 compared to GGN1 with 100% pure GGO (no solid component) in this study. These results suggest that the increase in the solid portion may turn on an angiogenic switch during tumor progression and potentiate the tumor invasiveness in ground-glass nodular lung adenocarcinoma. Finally, viral transcripts including JSRV were not detected in the tumors. Thus, viruses do not appear to be involved in the tumorigenesis of ground-glass nodular lung adenocarcinoma.

## Materials and Methods

### Patients and Samples

Nine patients with ground-glass nodular lung adenocarcinomas who were surgically treated at Samsung Medical Center (a 1,961-bed referral hospital in Seoul, South Korea) between January 2012 and December 2014 were included in the study. Tumor and normal tissues were snap frozen during surgical procedures and were stored in liquid nitrogen until use. Lung adenocarcinomas were pathologically classified based on the International Association for the Study of Lung Cancer/American Thoracic Society/European Respiratory Society classification^26^. The study was approved by the Institutional Review Board of Samsung Medical Center (IRB No. 2011-09-083) and all methods in this study were performed in accordance with the relevant guidelines and regulations. Written informed consent was obtained from each patient.

### Imaging Acquisition and Interpretation

Chest CT images were obtained with an 80- (LightSpeed Ultra; GE Healthcare, Mt. Prospect, IL, USA) or 16- (LightSpeed16; GE Healthcare) detector row CT scanner using the following parameters: detector collimation, 0.625 mm; field of view, 34.5 cm; beam pitch, 1.35 or 1.375; gantry speed, 0.6 s per rotation; 120 kVp; 150 to 200 mA; and section thickness, 1.25 mm for transverse images. Chest CT data were interfaced directly to a picture archiving and communication system (Path-Speed or Centricity 2.0; GE Healthcare) that displayed all of the image data on two monitors (1536 × 2048 matrix, 8-bit viewable grayscale, 60-foot-lambert luminescence). The monitors were adapted to view both mediastinal (width, 400 HU; level, 20 HU) and lung (width, 1500 HU; level, −700 HU) window images. A pure GGN was defined as a discrete pulmonary nodular abnormality with homogeneous attenuation that was not as high as that of the surrounding soft-tissue structures. A part-solid GGN was defined as a lesion containing both GGO and solid soft-tissue attenuation components. In all of the cases, the maximum diameter of the tumors (max*D*) and the largest dimension perpendicular (per*D*) to max*D* using both the lung and mediastinal windows were measured and the tumor disappearance rate and its quantification were calculated to assess tumor volume and the proportion of pure GGO (Supplementary Fig. S1)^40^.

### Isolation of Genomic DNA and RNA

Genomic DNA and RNA in tissues were purified using ALLPrep DNA/RNA Mini Kit (Qiagen). Genomic DNA concentration and purity were measured by a Nanodrop 8000 UV-Vis spectrometer (Thermo Scientific Inc.) and Qubit 2.0 Fluorometer (Life Technologies Inc.), respectively. To estimate DNA degradation, DNA median size was measured with a 2200 TapeStation Instrument (Agilent Technologies). For RNA, the concentration and purity was measured by Nanodrop and Bioanalyzer (Agilent Technologies).

### Targeted Exon Sequencing by Customized Cancer Panel

Genomic DNA from each sample was sheared by the Covaris S220 (Covaris, MA, USA) and used for the construction of a library using CancerSCAN probes and the SureSelectXT reagent kit (HSQ; Agilent Technologies, Santa Clara, CA, USA) according to the manufacturer’s protocol. CancerSCAN was designed to enrich the exons of 83 genes, covering 366.2 kb of the human genome (Supplementary Table S1)^41^. After enriched exon libraries were multiplexed, the libraries were sequenced on a HiSeq. 2500 sequencing platform (Illumina, San Diego, CA, USA). Briefly, a paired-end DNA sequencing library was prepared through gDNA shearing, end-repair, A-tailing, paired-end adaptor ligation, and amplification. After hybridization of the library with bait sequences for 27 h, the captured library was purified and amplified with an index barcode tag, and the library quality and quantity were measured. Sequencing of the exome library was performed using the 100 bp paired-end mode of the TruSeq Rapid PE Cluster kit and TruSeq Rapid SBS kit (Illumina).

### Variants Detection by Customized Cancer Panel

Sequence reads were mapped to the human genome (hg19) using Burrows-Wheeler Alignment tool. Duplicate read removal was performed using Picard and Samtools, and local alignment was optimized by The Genome Analysis Toolkit. Variant calling was only performed in targeted regions of CancerSCAN. Somatic variant calling of each tumor was based on the results of CancerSCAN of tumor tissue and RNA sequencing of tumor and normal tissues as targeted exon sequencing was only performed for tumor tissue. Among the variants which were detected by CancerSCAN, the variants which were detected only in the tumor tissue but not in the normal tissue by RNA sequencing were considered as true variants. To detect single nucleotide variants, we integrated the results of three types of variant caller, which increased the sensitivity. We used Pindel to detect indels^42^. All EGFR exon 19 deletions were considered as true variants.

### RNA Sequencing

The library construction for whole transcriptome analyses were performed using the TruSeq RNA sample preparation v2 kit (Illumina, USA). Isolated total RNA (2 µg) was used in a reverse transcription reaction with poly (dT) primers using the SuperScriptTM II reverse transcriptase (Invitrogen/Life Technologies, Grand Island, NY, USA) according to the manufacturer’s protocols. Briefly, a RNA sequencing library was prepared through cDNA amplification, end-repair, adenylate 3′ends, adapter ligation, and amplification. The quality and quantity of the library were measured with a Bioanalyzer and Qubit. Sequencing of the transcriptome library was performed using the 100 bp paired-end mode of the TruSeq Rapid PE Cluster kit and TruSeq Rapid SBS kit (Illumina, USA).

### RNA Sequencing Data Analysis

The reads from the FASTQ files were mapped against the hg19 human reference genome using TopHat version 2.0.6 (http://tophat.cbcb.umd.edu/). Raw read counts mapped onto genes were measured with the BAM format file by HTSeq version 0.6.1^43^. Then a total of 18,161 coding genes were subjected to measurement of the transcript abundance, and low expressed genes were filtered out using the criterion of a maximum read count >20 across all samples. Read counts were normalized by the trimmed mean of M-values normalization method. The differentially expressed genes were identified using the DESeq R package (www-huber.embl.de/users/anders/DESeq/). Gene set enrichment tests were performed using the GAGE R tool^44^. Clustering was performed by principal component analysis and hierarchical clustering. Gene ontology analyses were performed by DAVID (https://david.ncifcrf.gov/) and visualized by REVIGO (http://revigo.irb.hr/). The TopHat-fusion tool was used for the detection of fusion gene loci.

### Detection of Viral Genomes

A total of 6,251 viral genomes were obtained from ftp://ftp.ncbi.nih.gov/refseq/release/viral/viral.1.1.genomic.fna.gz. RNA-Seq reads were mapped to the reference human genome (hg19) using TopHat. Then unmapped reads were chosen and were converted into the FASTQ format. They were mapped with zero mismatch to 6,251 viral genomes using the Bowtie tool (http://bowtie-bio.sourceforge.net). Mapped read counts were compared between normal and tumor samples.

### RT-PCR Validation

The fusion genes detected by RNA sequencing were validated by reverse transcriptase-polymerase chain reaction (RT-PCR). Fresh-frozen tumor tissues were homogenized with the Qiagen’s TissueLayser, using 5 mm stainless steel beads according to the manufacturers’ instructions. Total RNA was extracted from fresh-frozen tumor tissues using RNeasy Mini Kit and RNase-free DNase set (Qiagen, Valencia, CA). cDNA was synthesized from 1 µg of total RNA using the SuperScriptTM II Reverse Transcriptase (Invitrogen, Carlsbad, MD) and random primers (250 ng). After cDNA synthesis, RT-PCR was performed using primer flanking the gene fusion junction. The PCR primer sequences used are listed in Supplementary Table S2.

### Statistical Analysis

Categorical and continuous variables are presented as number (%) and median (interquartile range [IQR]), respectively. Categorical variables were compared using Pearson’s χ^2^ test or Fisher’s exact test and continuous variables were analyzed using the Mann-Whitney U test. All tests were two-sided and a P value < 0.05 was considered statistically significant. All statistical analyses were performed using SPSS Statistics for Windows, version 23.0 (IBM Corp., Armonk, NY, USA).

### Data Availability

All data generated or analysed during this study are included in this published article and its Supplementary Information files.

## Electronic supplementary material

Supplementary information

supplementary Table S3

supplementary Table S4

supplementary Table S5
