# Supplementary material for: Genomic alterations of ground-glass nodular lung adenocarcinoma
Source: Sci Rep. 2018 May 16;8:7691. doi: 10.1038/s41598-018-25800-2 (PMC5955945; doi:10.1038/s41598-018-25800-2)

Genomic alterations of ground-glass nodular lung adenocarcinoma

Hyun Lee[1], Je-Gun Joung[2], Hyun-Tae Shin[2], Duk-Hwan Kim[3], Yujin Kim[3], Hojoong Kim[1], O Jung Kwon[1], Young Mog Shim[4], Ho Yun Lee[5], Kyung Soo Lee[5], Yoon-La Choi[6], Woong-Yang Park[2], D. Neil Hayes[7], Sang-Won Um[1]

Supplementary information

Supplementary Figure S1. The proportions of solid and pure ground-glass opacity in GGN7. The proportions of solid and pure ground-glass opacity were determined using the tumor-shadow disappearance ratio on a computed tomography scan.

Supplementary Figure S2. The IGV visualization of fusion genes detected by RNA sequencing.

(**a**) MED13L → TDRD3 in GGN3, (**b**) SAMD12 → TAF2 in GGN4, (**c**) CEP350 → TOP2A and (**d**) TADA2A→ MMP9 in GGN8, and (**e**) TMEM243 → DMTF1 in GGN9

Supplementary Figure S3. Validation of fusion genes by RT-PCR. The image of full-length gels was presented without grouping of images by cropping gels.

Supplementary Table S1. List of genes detectable by CancerSCAN v1.1

Supplementary Table S2. List of primer sequences used for RT-PCR.

Supplementary Table S3. List of somatic changes detected by targeted exon sequencing.

Supplementary Table S4. List of gene expression profiles of ground-glass nodular lung adenocarcinoma.

Supplementary Table S5. List of differentially expressed genes between lung adenocarcinoma tissue and normal tissue.

Supplementary Table S1. List of genes detectable by CancerSCAN v1.1

SNV, small Indel and CNV				
ABL1	BRCA2	FGFR2	KIT	PTCH1
AKT1	CDH1	FGFR3	KRAS	PTCH2
AKT2	CDK4	FLT3	MDM2	PTEN
AKT3	CDK6	GNA11	MET	PTPN11
ALK	CDKN2A	GNAQ	MLH1	RB1
APC	CSF1R	GNAS	MPL	RET
ARID1A	CTNNB1	HNF1A	MTOR	ROS1
ARID1B	DDR2	HRAS	NF1	SMAD4
ARID2	EGFR	IDH1	NOTCH1	SMARCB1
ATM	EPHB4	IDH2	NPM1	SMO
ATRX	ERBB2	IGF1R	NRAS	SRC
AURKA	ERBB3	ITK	NTRK1	STK11
AURKB	ERBB4	JAK1	PDGFRA	SYK
BCL2	EZH2	JAK2	PDGFRB	TOP1
BRAF	FBXW7	JAK3	PIK3CA	TP53
BRCA1	FGFR1	KDR	PIK3R1	VHL
Translocation				
ALK	EWSR1	ROS1	RET	TMPRSS2
		TERT		

CancerSCAN targets somatic mutations of 80 genes all exon (SNV, small Indel and CNV) and 5 genes translocation. The target size is 366.231 Kb.

SNV, single nucleotide variant; CNV, copy number variant.

Supplementary Table S2. List of PCR primer sequences used for RT-PCR

GGN	Gene	Primer No.	Sequence (5'-3')		product size, bp	Annealing Temp, ℃
			Forward primer	Reverse primer		
GGN3	MED13L → TDRD3	1	GCACCAGAGGA GCTAAAGCA	GATCGTAGGTG CCTTCTTCC	154	57
GGN4	SAMD12 → TAF2	2	AAAGGAACTCC CAAGCGACT	CGGAGCTGATA CTGCCACAT	213	57
		3	GAAGCTGAGAC GGCTAAGGA	CGGAGCTGATA CTGCCACAT	186	57
GGN8	CEP350 → TOP2A	4	GGAGGCGACAC TCTCAGGTA	CCACCCTTGGA TGTAGCAAT	196	57
	TADA2A → MMP9	5	CTCGAGCAGAT TTCATTGAGG	GCCCTCAAAGG TTTGGAATC	218	57
GGN9	TMEM243→DMTF1	6	TGCAGTCATGG TCACTTCAA	TAAGTGGCTTG GGGAAAGTG	152	57
		7	TGCAGTCATGG TCACTTCAA	CAGTCGTGGAC CTGAGACCT	196	57

GGN, ground glass nodule; Temp, temperature; No., number; PCR, polymerase chain reaction; RT-PCR, reverse transcription-polymerase chain reaction.

Supplementary Figure S1. The proportions of solid and pure ground-glass opacity in GGN7. The proportions of solid and pure ground-glass opacity were determined using the tumor-shadow disappearance ratio on a computed tomography scan.

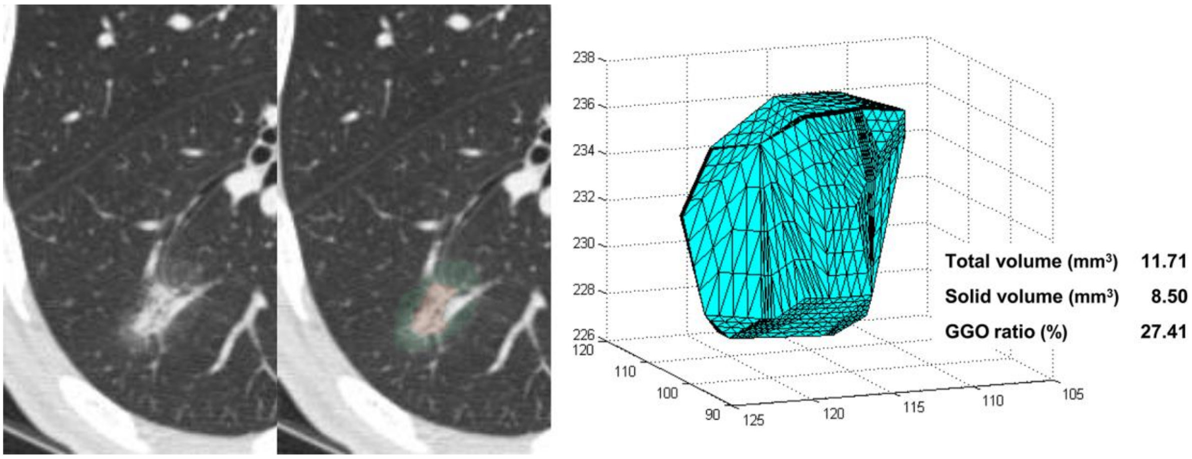

Supplementary Figure S2. The IGV visualization of fusion genes detected by RNA sequencing.

(**a**) MED13L → TDRD3 in GGN3, (**b**) SAMD12 → TAF2 in GGN4, (**c**) CEP350 → TOP2A and (**d**) TADA2A→ MMP9 in GGN8, and (**e**) TMEM243 → DMTF1 in GGN9

A

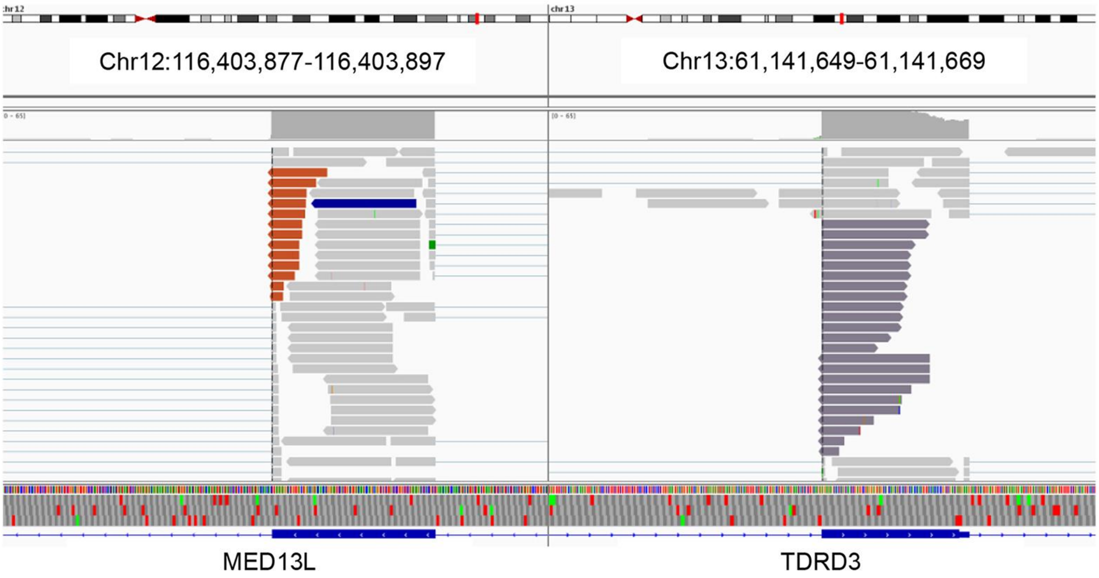

B

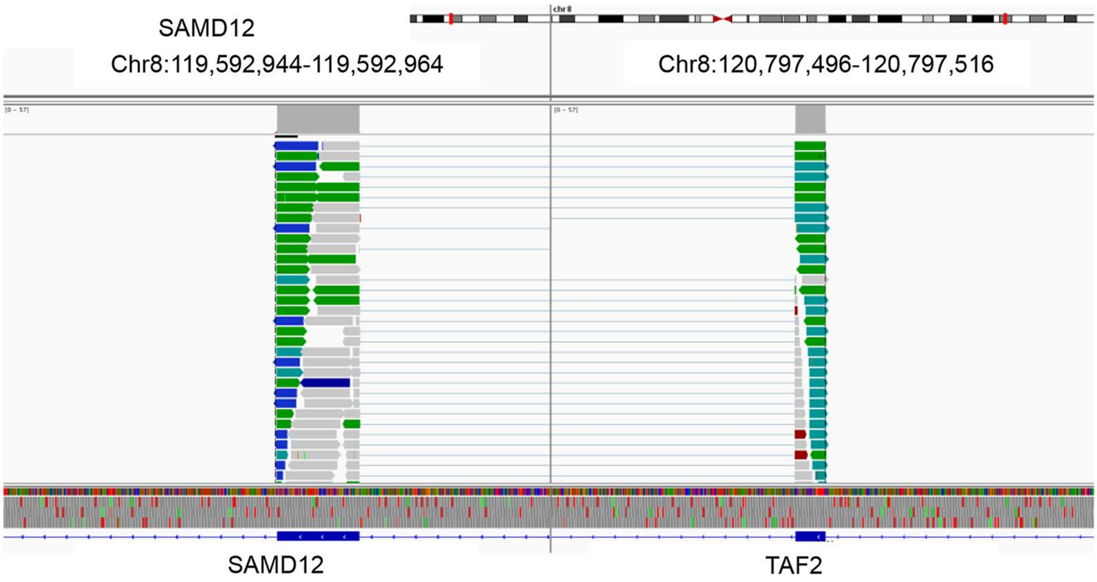

C

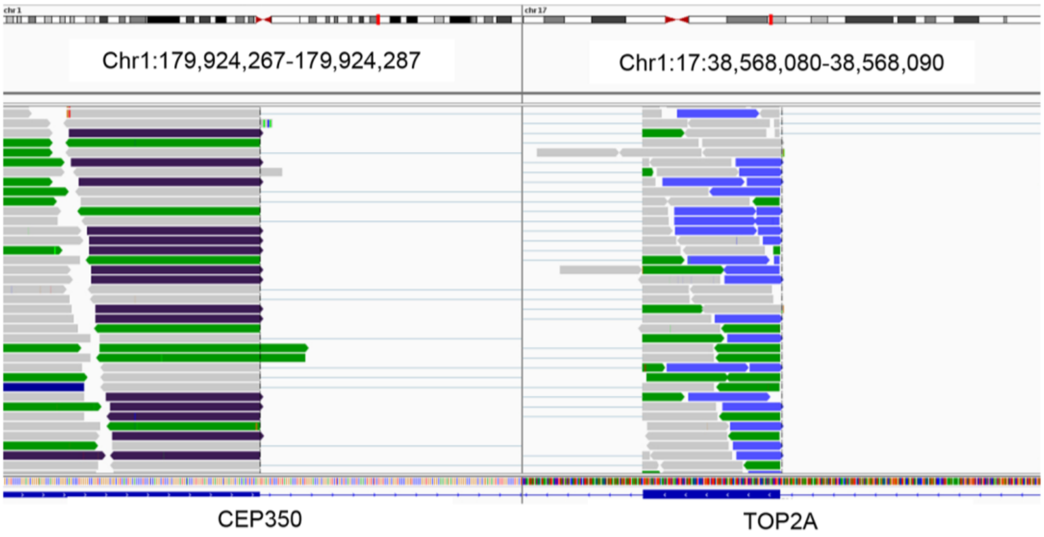
Chr1:179,924,267-179,924,287
Chr1:17:38,568,080-38,568,090
CEP350
TOP2A

D

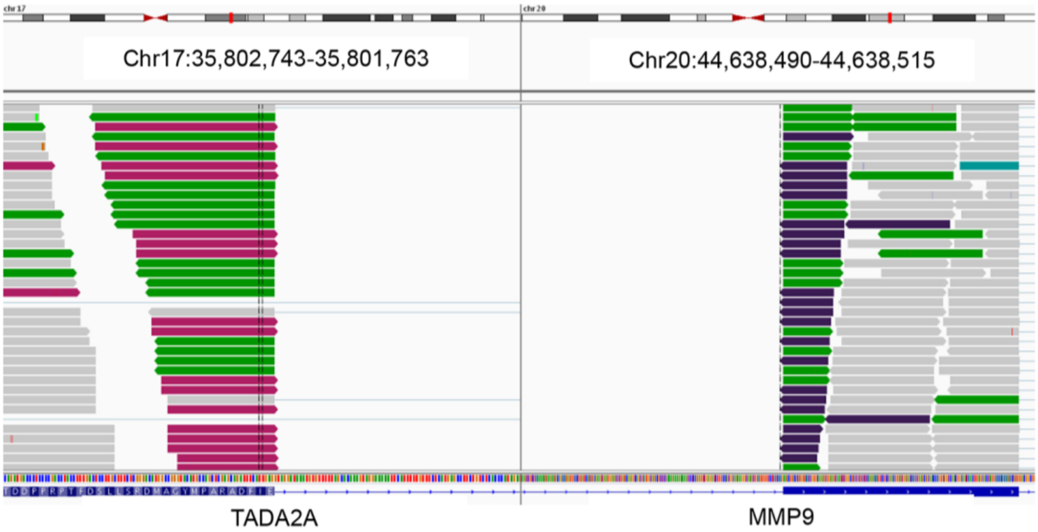
Chr17:35,802,743-35,801,763
Chr20:44,638,490-44,638,515
TADA2A
MMP9

E

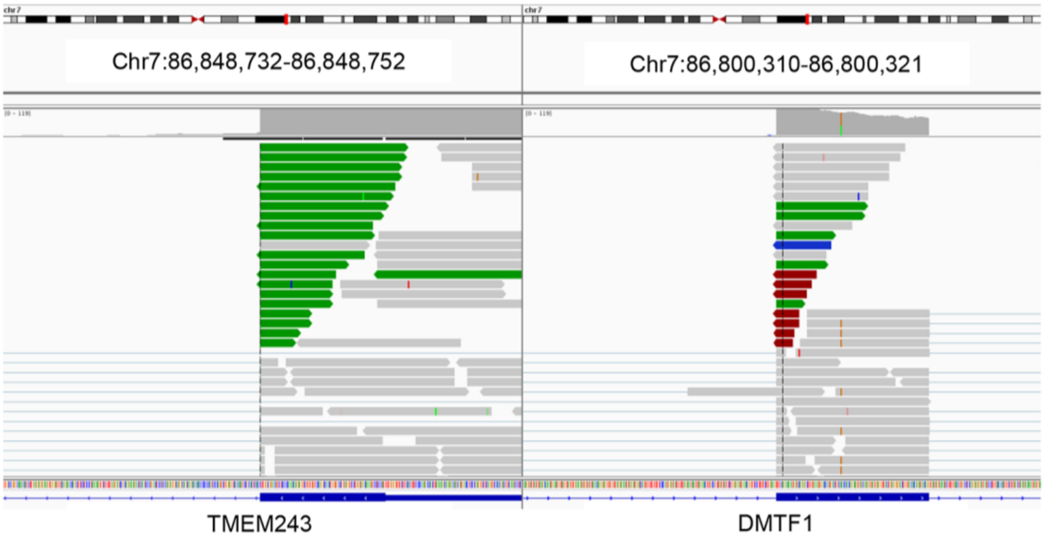
Chr7:86,848,732-86,848,752
Chr7:86,800,310-86,800,321
TMEM243
DMTF1

Supplementary Figure S3. Validation of fusion genes by RT-PCR. The image of full-length gels was presented without grouping of images by cropping gels.

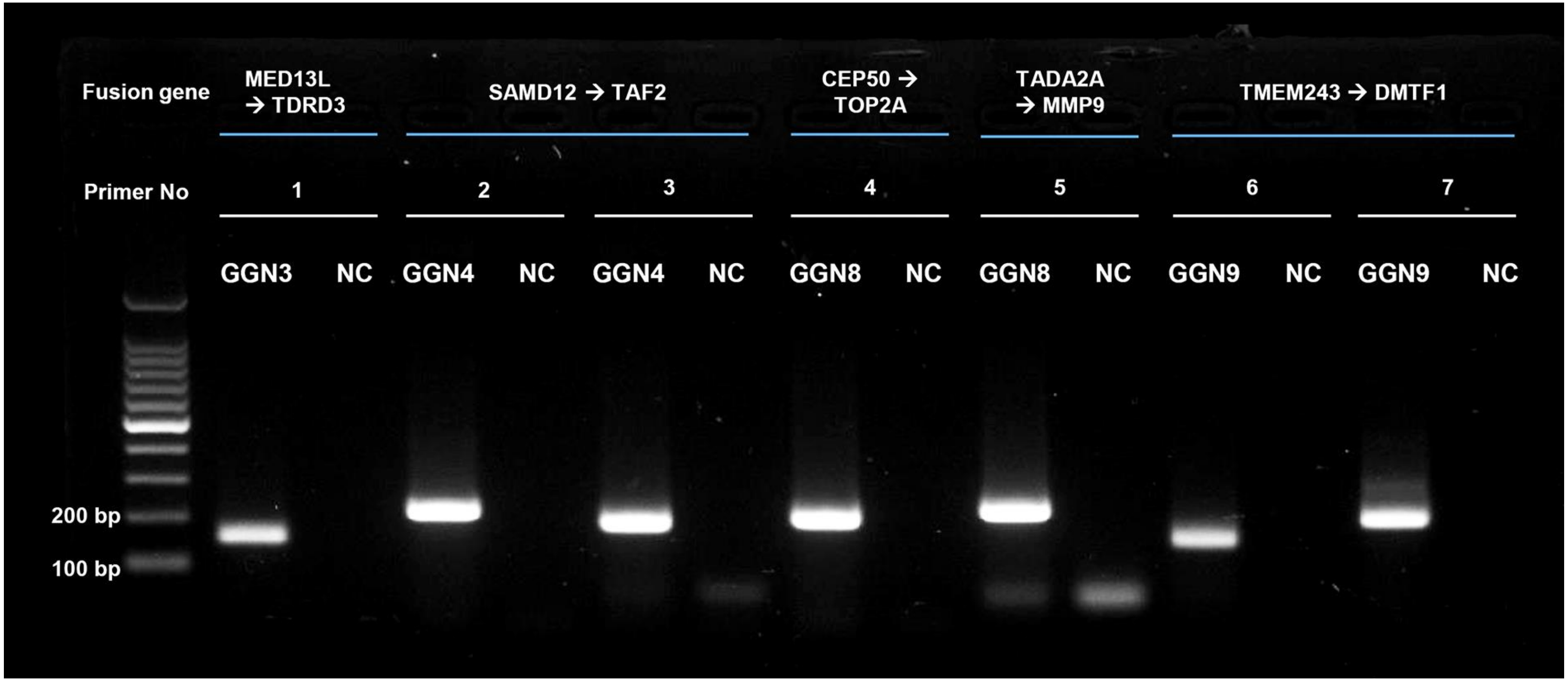

Supplement: Supplementary file 1 — Supplementary information [file 41598_2018_25800_MOESM1_ESM.pdf]
